# High temperature AlInP X-ray spectrometers

**DOI:** 10.1038/s41598-019-48394-9

**Published:** 2019-08-21

**Authors:** S. Zhao, S. Butera, G. Lioliou, A. B. Krysa, A. M. Barnett

**Affiliations:** 1 Space Research Group, Department of Engineering and Design, School of Engineering and Informatics, University of Sussex, Falmer, Brighton BN1 9QT UK; 2 EPSRC National Epitaxy Facility, University of Sheffield, Mappin Street, Sheffield, S1 3JD UK

**Keywords:** Electronic devices, Astronomical instrumentation

## Abstract

Two custom-made Al_0.52_In_0.48_P p^+^-i-n^+^ mesa photodiodes with different diameters (217 µm ± 15 µm and 409 µm ± 28 µm) and i layer thicknesses of 6 µm have been electrically characterised over the temperature range 0 °C to 100 °C. Each photodiode was then investigated as a high-temperature-tolerant photon counting X-ray detector by connecting it to a custom-made low-noise charge-sensitive preamplifier and illuminating it with an ^55^Fe radioisotope X-ray source (Mn Kα = 5.9 keV; Mn Kβ = 6.49 keV). At 100 °C, the best energy resolutions (*full width at half maximum* at 5.9 keV) achieved using the 217 µm ± 15 µm diameter photodiode and the 409 µm ± 28 µm diameter photodiode were 1.31 keV ± 0.04 keV and 1.64 keV ± 0.08 keV, respectively. Noise analysis of the system is presented. The dielectric dissipation factor of Al_0.52_In_0.48_P was estimated as a function of temperature, up to 100 °C. The results show the performance of the thickest Al_0.52_In_0.48_P X-ray detectors so far reported at high temperature. The work has relevance for the development of novel space science instrumentation for use in hot space environments and extreme terrestrial applications.

## Introduction

X-ray spectroscopy is a key technology for many space science applications, including *in situ* planetary and comet analysis^1,2^, planetary remote sensing^3,4^, and observation of solar activities^5,6^. However, the temperature in space environments can vary greatly (e.g. −50 °C to +70 °C at the surface of Mercury^7^). When using narrow bandgap semiconductor X-ray detectors (e.g. Si, which has a bandgap energy of 1.12 eV at room temperature^8^) in a high temperature (>20 °C) environment, cooling systems are required to reduce the detector’s leakage current^9,10^ and to mitigate radiation damage effects^9^. Wide bandgap semiconductors have a lower intrinsic carrier concentration than narrow bandgap semiconductors due to the dependency of the intrinsic carrier concentration on the bandgap energy^8^, consequently cooling systems may be eliminated when wide bandgap semiconductor detectors used. This brings the advantages of lower instrument and spacecraft mass, volume, power consumption, and cost.

As such, many wide bandgap semiconductor materials such as SiC^11,12^, GaAs^13,14^, AlGaAs^15,16^, and InGaP^17,18^ have been intensively studied for high temperature X-ray detection applications. One of the other many interesting materials for such application is Al_0.52_In_0.48_P. Al_0.52_In_0.48_P is a wide bandgap semiconductor (*E*_*g*_** = **2.31 eV at room temperature^19^) which is nearly lattice matched with GaAs. Al_0.52_In_0.48_P has also been investigated for its use in other applications including solar cells^20^ and undersea optical communications^21^. Because of the intrinsic advantages of Al_0.52_In_0.48_P, e.g. high X-ray linear absorption coefficient (at 5.9 keV, 1302 cm^−1^ for Al_0.52_In_0.48_P, 837 cm^−1^ for GaAs, 640 cm^−1^ for Al_0.8_Ga_0.2_As, and 346 cm^−1^ for 4H-SiC^22^) and moderate electron-hole pair creation energy (at 20 °C, 5.34 eV for Al_0.52_In_0.48_P^23^ cf. 7.8 eV for 4 H-SiC^24^), Al_0.52_In_0.48_P has started to be investigated as a potentially useful material for X-ray detection^25–27^.

The energy resolution of an X-ray spectrometer, employing a semiconductor photodiode detector coupled to a charge-sensitive preamplifier, is commonly limited by the parallel white electronic noise from the detector and the preamplifier at high temperatures^28^. However, using a high-quality wide bandgap semiconductor photodiode detector and a low-noise charge-sensitive preamplifier^11,13,27^, excellent energy resolutions can be achieved. By far, Bertuccio *et al*. have reported the best high temperature X-ray spectrometer energy resolution: 233 eV *FWHM* at 5.9 keV at 100 °C. This was achieved using a 4 H-SiC Schottky diode detector^11^ and an ultra-low-noise charge-sensitive preamplifier. In comparison, for Al_0.52_In_0.48_P, the best high temperature (100 °C) energy resolution so far reported is 1.57 keV *FWHM* at 5.9 keV^27^. This result was achieved using the first non-avalanche Al_0.52_In_0.48_P X-ray photodiode ever reported^27^. It had a 2 μm thick i layer. Room temperature results have also been reported using 6 µm i layer Al_0.52_In_0.48_P photodiodes^29^.

In the new work reported in the current article, two 6 µm i layer Al_0.52_In_0.48_P photodiodes are extensively characterised at temperatures from 0 °C to 100 °C as part of efforts to develop photon counting X-ray spectrometers for future space science missions.

## Results

### Capacitance measurements as functions of applied reverse bias

**Figure 1:**
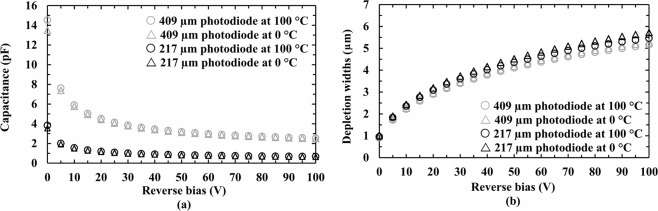
(**a**) Measured capacitances and (**b**) calculated depletion widths as functions of applied reverse bias for the 217 µm diameter photodiode (100 °C, black open circles; 0 °C, black open triangles) and 409 µm diameter photodiode (100 °C, grey open circles; 0 °C, grey open triangles).

The capacitance of each device as a function of applied reverse was measured at different temperatures, using an HP 4275A Multi Frequency LCR meter and a Keithley 6487 Picoammeter/Voltage Source. In order to control the temperature, each photodiode was installed in a light-tight custom-made aluminium test harness inside a dry N_2_ filled TAS Micro MT environmental Test Chamber (relative humidity <5%). The LCR meter had an AC test signal with 60 mV r.m.s amplitude and 1 MHz frequency. The Keithley Picoammeter/Voltage Source was used to reverse bias the photodiodes up to 100 V in increments of 1 V. The capacitance measurements were automated using National Instruments’ Labview software. Before starting the measurements at each temperature, each device was allowed to stabilise for 30 min to achieve thermal equilibrium. The devices’ capacitances as functions of applied reverse bias were measured from 100 °C to 0 °C, with a decrement step of 20 °C. Because the photodiodes were packaged in a TO-5 can, the capacitance between an empty pin on the package (a pin without a wire-bonded device) and the common pin of the package was used to estimate the capacitance contribution of the packaging. The package capacitance was found to be temperature independent within the investigated temperature range. The capacitance of each photodiode was calculated by subtracting the capacitance of the package (0.80 pF ± 0.05 pF) from the total measured capacitance of the packaged device. The devices’ capacitances (packaging subtracted), and the calculated depletion widths of the photodiodes as functions of applied reverse bias at 100 °C and 0 °C, are presented in Fig. 1. At the highest investigated temperature (100 °C) and reverse bias (100 V), the capacitances of the 217 µm diameter photodiode and the 409 µm diameter photodiode were found to be 0.67 pF ± 0.07 pF (corresponding capacitance density of 2.2 nF/cm^2^ ± 0.3 nF/cm^2^) and 2.54 pF ± 0.09 pF (corresponding capacitance density of 1.9 nF/cm^2^ ± 0.2 nF/cm^2^), respectively. The uncertainties reflect an experimental repeatability uncertainty (±0.07 pF) and the measurement uncertainty (~ 0.1%) of the LCR meter. At high reverse biases (≥80 V), the reduction in the capacitance with increased applied reverse bias was found to be negligible within the uncertainties. Therefore, the photodiodes were considered to be fully depleted at 80 V reverse bias. The calculated depletion widths of the 217 µm diameter photodiode at 100 V reverse bias were found to be 5.5 µm ± 0.7 µm at 100 °C and 5.7 µm ± 0.8 µm at 0 °C, respectively. At 100 V reverse bias, the calculated depletion widths of the 409 µm diameter photodiode were respectively found to be 5.1 µm ± 0.5 µm at 100 °C and 5.2 µm ± 0.5 µm at 0 °C. The uncertainties in the depletion widths were calculated by taking into account the uncertainties in the diameters, the uncertainties in the capacitance measurements, and the Debye length^30^.

### Leakage currents as functions of applied reverse bias measurements

**Figure 2:**
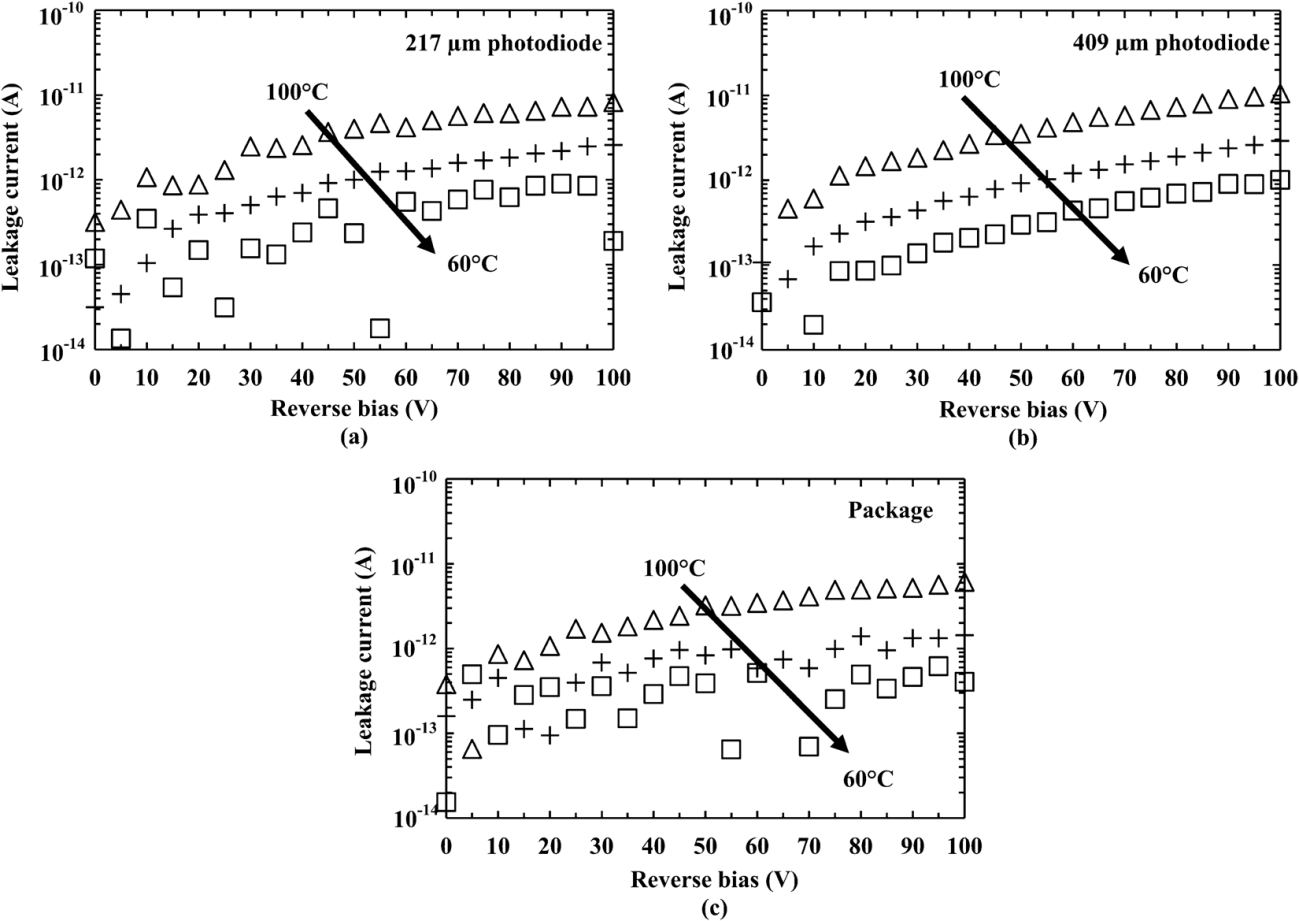
Leakage currents as functions of reverse bias for (**a**) packaged 217 µm diameter detector (including package leakage), (**b**) packaged 409 µm diameter detector (including package leakage), and (**c**) the measured leakage current contribution in (a) and (b) from the package itself (as measured using a package pin without a wire-bonded device). In each case the measurements are shown at temperatures of 100 °C, 80 °C, and 60 °C. At temperatures <60 °C, the currents were too small to be reliable measured using the available experimental set up.

The leakage currents of the two detectors were measured as functions of applied reverse bias across the temperatures range 100 °C to 0 °C using the same climatic procedure as was employed for the capacitance measurements. A Keithley 6487 Picoammeter/Voltage Source was used to reverse bias the devices from 0 V to 100 V, in steps of 1 V, and measure the resultant current. The measurements were automated using National Instruments’ Labview software. The leakage current of the package, i.e. the leakage current between an empty pin (a pin without a wire-bonded device) and the common pin of the package, was also measured. The results showed that the leakage currents of the package itself and the packaged devices (including the leakage current of the package and the photodiodes) increased with increasing temperature, as presented in Fig. 2. The leakage current of the package was found to be the dominant contributor to the leakage currents for both packaged devices. As such, comparable leakage currents were measured for both devices, at each applied reverse bias and temperature. At the highest investigated temperature (100 °C) and 100 V reverse bias (electric field strength = 167 kV/cm), the leakage currents of the packaged 217 µm diameter device (including package leakage), and the packaged 409 µm diameter device (including package leakage) were found to be 8.3 pA ± 0.4 pA and 10.5 pA ± 0.4 pA, respectively. The leakage current contribution to these values from the package was 6.1 pA ± 0.4 pA. At temperatures <60 °C the leakage currents were smaller than the uncertainty of the measuring system (±0.4 pA).

### Photon counting X-ray spectroscopy

To characterise the detectors’ responses to illumination with X-rays, each of the detectors was in turn coupled to a custom-made low-noise charge-sensitive feedback resistorless preamplifier (similar to that reported in ref.^31^) and illuminated with an ^55^Fe radioisotope X-ray source (Mn Kα = 5.9 keV; Mn Kβ = 6.49 keV; activity = 171 MBq; active area = 28.27 mm^2^). A wire-ended packaged silicon JFET (2N4416A, capacitance = 2 pF at room temperature) was used as the input transistor of the preamplifier. Each system was installed in a dry N_2_ filled TAS Micro MT Environmental Test Chamber (relative humidity <5%). The output of each preamplifier was connected to an ORTEC 572A shaping amplifier, and the output of the shaping amplifier was connected to an ORTEC EASY-MCA 8k multi-channel analyser (MCA). The ^55^Fe radioisotope X-ray source was placed on a PTFE custom holder 5 mm above the detectors. To reduce the count rate seen with the 409 µm diameter photodiode so that it was approximately equal to that seen with the 217 µm diameter photodiode, a 0.23 mm thick polytetrafluoroethylene (PTFE) attenuator was inserted into the 5 mm gap for those measurements. In order to ensure thermal equilibrium at each temperature, the systems were allowed to stabilise for 30 min at each temperature prior to commencing accumulation of the spectra. Different shaping times (0.5 µs, 1 µs, 2 µs, 3 µs, 6 µs, and 10 µs) and reverse biases (0 V, 20 V, 40 V, 60 V, and 100 V) were used across the temperature range (100 °C to 0 °C), to investigate the performances of the systems. Each spectrum had a live time limit of 240 s.

**Figure 3:**
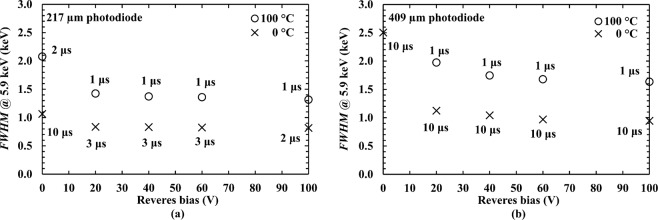
Measured best energy resolution (*FWHM* at 5.9 keV) achieved using (**a**) the 217 µm diameter photodiode and (**b**) the 409 µm diameter photodiode as functions of applied reverse bias at each investigated temperature and at the optimum shaping time, 100 °C (open circles), and 0 °C (× symbols).

The obtained spectra were energy calibrated using the position of the zero energy noise peak and the centroid channel number of the fitted Mn Kα at 5.9 keV for each spectrum, as points of known energies on MCA’s charge scale. The energy resolution (as quantified by the *FWHM* at 5.9 keV) of the system as a function of applied reverse bias, at the highest (100 °C) and lowest (0 °C) investigated temperatures is shown in Fig. 3. The ^55^Fe X-ray photopeak of the spectrum accumulated using the 409 µm diameter photodiode could not be resolved from the zero noise peak when no reverse bias was applied at 100 °C, due to the relatively large capacitance of the photodiode (15 pF at 0 V at 100 °C). The *FWHM* at 5.9 keV of both spectrometers were found to be decreased with increasing reverse bias. This may be explained due to the reduced capacitance of the photodiodes (see Fig. 1) and the improved charge collection, with increasing reverse bias. At the highest investigated reverse bias (100 V) and the highest investigated temperature (100 °C), the best energy resolutions achieved with the 217 µm diameter photodiode (active area of 0.04 mm^2^) and the 409 µm diameter photodiode (active area of 0.13 mm^2^) were found to be 1.31 keV ± 0.04 keV and 1.64 keV ± 0.08 keV, respectively.

**Figure 4:**
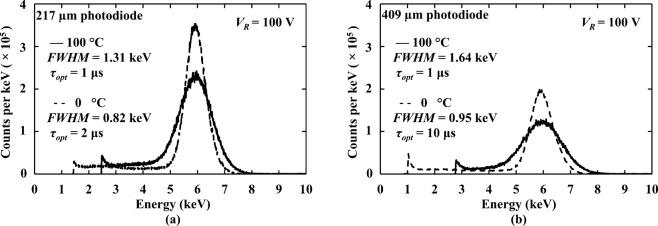
^55^Fe X-ray spectrum obtained with (**a**) 217 µm diameter photodiode and (**b**) 409 µm diameter photodiode at 100 V reverse bias, at a temperature of 100 °C (solid line) and 0 °C (dashed line), at the optimum shaping time.

The spectra obtained with the spectrometers at 100 °C and 0 °C, with the detectors reverse biased at the 100 V are shown in Fig. 4. The number of counts is not comparable between the different diameter photodiodes on an area normalised basis because of the presence of the 0.23 mm thick PTFE absorber in the case of the 409 µm diameter photodiode. The energy resolutions (*FWHM* at 5.9 keV) achieved with these Al_0.52_In_0.48_P detector X-ray spectrometers were not as good as those achieved using high-quality 4 H-SiC detectors (70 µm thick epitaxial layer; area of 0.04 mm^2^) and ultra-low-noise preamplifier electronics (233 eV *FWHM* at 5.9 keV at 100 °C)^11^. However, they are better than has been previously reported at 100 °C with other Al_0.52_In_0.48_P detectors (comparing the 217 µm diameter detector with a previously reported 2 μm thick Al_0.52_In_0.48_P device of the same size) (1.31 keV cf. 1.57 keV *FWHM* at 5.9 keV)^27^, and comparable to the results obtained with In_0.5_Ga_0.5_P photodiodes at 100 °C (5 μm thick i layer; 200 µm diameter; 1.27 keV *FWHM* at 5.9 keV)^18^. They also have better energy resolution than the best reported Al_0.8_Ga_0.2_As photodiodes (1 μm thick i layer; 200 µm diameter; 2.2 keV *FWHM* at 5.9 keV) at 90 °C^15^.

### Noise analysis

Ideally, the energy resolution of a non-avalanche photodiode based X-ray spectrometer is only limited by the Fano noise; the Fano noise depends on the electron-hole pair creation energy of the semiconductor, the Fano factor, and the incident X-ray photon energy^8^. The Fano-limited energy resolution (*FWHM*_Fano_) at 5.9 keV of Al_0.52_In_0.48_P can be estimated to be 145 eV at 20 °C, assuming a Fano factor of 0.12 and given an electron-hole pair creation energy of 5.34 eV^23^. However, the experimental energy resolutions of the X-ray spectrometers reported in this present work were further degraded by electronic noise^28,32^.

**Figure 5:**
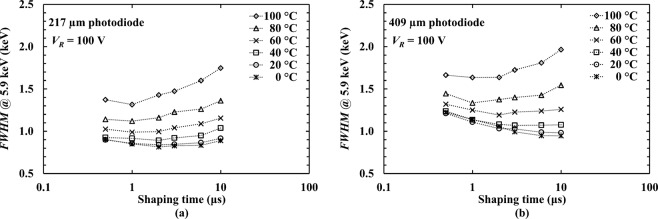
Measured *FWHM* at 5.9 keV as functions of shaping time for the Al_0.52_In_0.48_P photodiodes based spectrometers (**a**) 217 μm diameter photodiode (**b**) 409 μm diameter photodiode across the temperature range of 0 °C to 100 °C. The dotted lines are guides for the eyes only.

The electronic noise components in a semiconductor photodiode X-ray spectrometer are series white noise (including induced gate current noise), parallel white noise, 1/*f* series noise, and dielectric noise. Among these noise components, 1/*f* series noise and dielectric noise are independent of shaping time^32^. The series white noise including induced gate current noise is related to the total capacitance at the input of the preamplifier (e.g. stray capacitance, feedback capacitance, the capacitance of the photodiode, and the capacitance of the input JFET)^28^; it increases with decreasing shaping time. The parallel white noise is related to the total leakage current at the input of the preamplifier (leakage current of the photodiode and the leakage current of the input JFET); it increases with increasing shaping time. Therefore, the combination of the series white noise and the parallel white noise can be minimised by selecting an optimum shaping time. The measured *FWHM* at 5.9 keV of the Al_0.52_In_0.48_P X-ray spectrometers as functions of shaping time at each investigated temperature at 100 V reverse bias, are shown in Fig. 5. In this figure, an improvement can be seen in *FWHM* at 5.9 keV for both X-ray spectrometers at each investigated shaping time when the temperature decreased from 100 °C to 40 °C. The improvement in *FWHM* for both X-ray spectrometers was comparatively slight when the temperature decreased from 40 °C to 0 °C.

In order to achieve the best energy resolution at each investigated temperature, it was necessary to select different shaping times at different temperatures. For the spectrometer with the 217 μm diameter detector, the best available shaping times were 2 μs for temperatures of 0 °C to 40 °C, and 1 μs for 60 °C to 100 °C. For the spectrometer with the 409 μm diameter detector, the best available shaping times were 10 μs for temperatures of 0 °C and 20 °C, 3 μs for 40 °C, 2 μs for 60 °C, and 1 μs for 80 °C and 100 °C.

**Figure 6:**
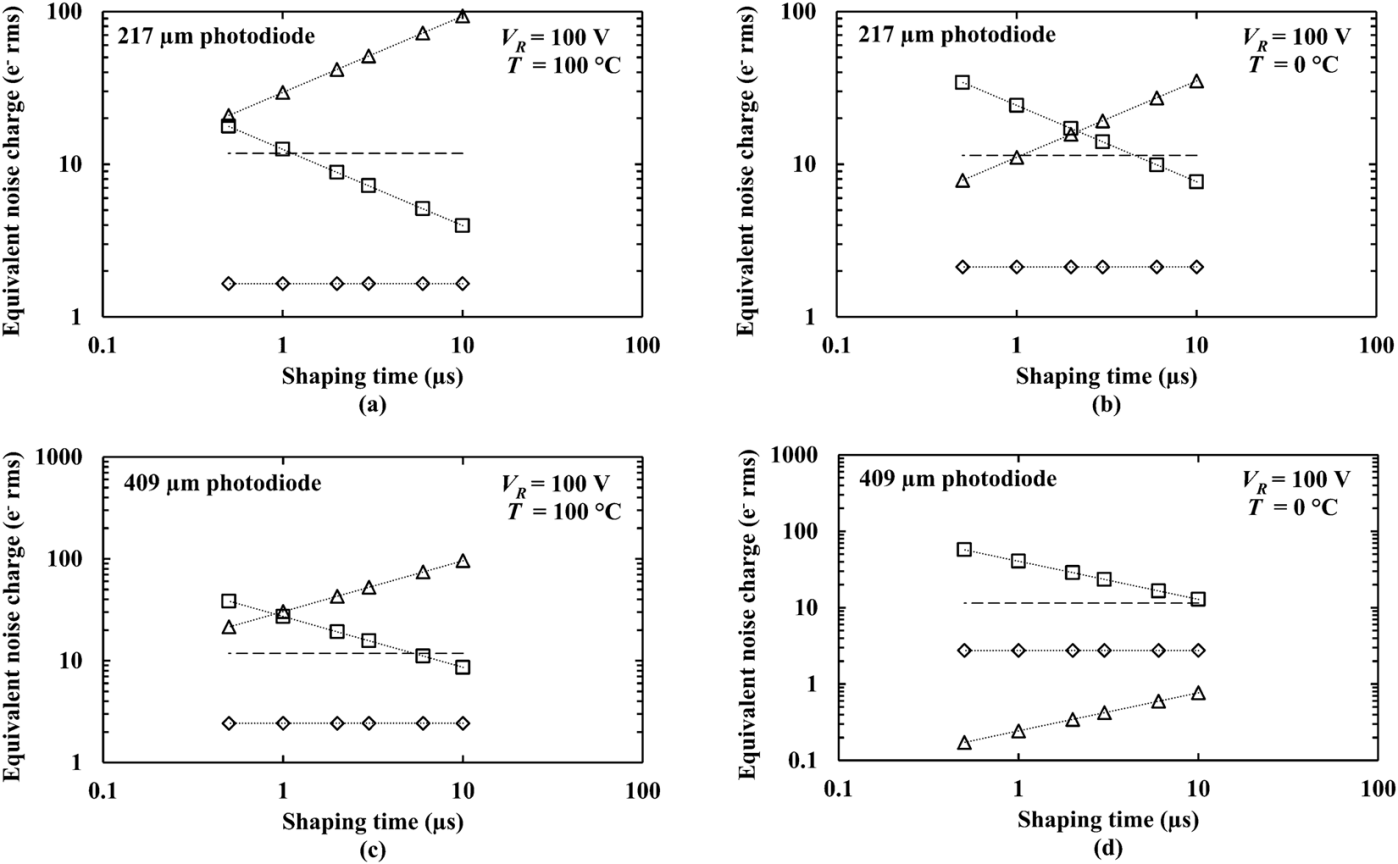
Equivalent noise charge as a function of shaping time for the Al_0.52_In_0.48_P photodiodes connected to the custom low-noise charge-sensitive preamplifier at 100 V reverse bias, at temperature of 100 °C and 0 °C; (**a**,**b**) for 217 μm diameter photodiode at 100 °C and 0 °C, and (**c**,**d**) for 409 µm diameter photodiode at 100 °C and 0 °C. Series white noise including induced gate current noise (open squares), parallel white noise (open triangles), Fano noise (dash line), and 1/*f* series noise (open diamonds). The dotted lines are guides for the eyes only.

At each investigated temperature, the total leakage current and the total capacitance at the input of the preamplifier can be estimated by applying a multidimensional unconstrained nonlinear minimisation to the measured *FWHM* at 5.9 keV as a function of shaping time for both X-ray spectrometer at 100 V reverse bias (see Fig. 5), the details are described in ref.^33^. The series white noise (including induced gate current), parallel white noise, and 1/*f* series noise were calculated as described in ref.^32^ using the estimated total leakage current and the total capacitance at the input of the preamplifier at each temperature. The calculated noise contributions of the series white noise (including induced gate current), parallel white noise, 1/*f* series noise, along with the estimated Fano noise as functions of shaping time for both X-ray spectrometers, at 100 V reverse bias, at the highest investigated temperature (100 °C) and the lowest investigated temperature (0 °C), are shown in Fig. 6.

At the highest investigated bias (100 V), at the available optimum shaping time at each temperature, the energy resolutions of both photodiodes spectrometers were found to be improved with decreasing temperature (see Fig. 5), e.g. *FWHM* at 5.9 keV of 1.31 keV at 100 °C cf. 0.82 keV at 0 °C for the 217 μm photodiode spectrometer; *FWHM* at 5.9 keV of 1.64 keV at 100 °C cf. 0.95 keV at 0 °C for the 409 μm photodiode spectrometer. Comparing 100 °C with 0 °C, the parallel white noise was significantly reduced, as shown in Fig. 6. Therefore, much of the improvement in energy resolution at low temperatures stemmed from the reduced parallel white noise (e.g. 93 e^−^ rms at 100 °C cf. 35 e^−^ rms at 0 °C for 217 μm photodiode spectrometer at a shaping time of 10 μs; compared with 96 e^−^ rms at 100 °C and 1 e^−^ rms at 0 °C for 409 μm photodiode spectrometer at the same shaping time). The majority of the parallel white noise at high temperature came from the leakage current of the preamplifier input JFET (88 e^−^ rms, at 100 °C) rather than the detectors. This emphasises the importance of developing new high temperature tolerant preamplifier electronics based on wide bandgap semiconductors^32^.

**Figure 7:**
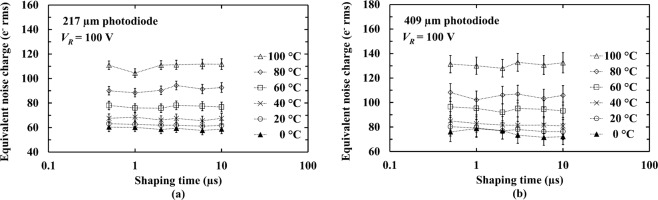
Equivalent noise charge of the dielectric noise as a function of shaping time for the (**a**) 217 μm and (**b**) 409 μm diameter Al_0.52_In_0.48_P photodiode X-ray spectrometers at 100 V reverse bias, at different temperatures. 100 °C (open triangles); 80 °C (open diamonds); 60 °C (open squares); 40 °C (×symbols); 20 °C (open circles); 0 °C (dark triangles). The dotted lines are guides for the eyes only.

The energy resolution of a photodiode X-ray spectrometer may also be affected by incomplete charge collection noise. However, according to results reported previously^26^, the incomplete charge collection noise of these particular photodiodes was negligible at high reverse biases (≥80 V). Therefore, at such detector reverse biases, the dielectric noise of the spectrometer can be calculated by subtracting in quadrature the calculated series white noise (including induced gate current noise), parallel white noise, 1/*f* series noise, and the predicted Fano noise, from the measured *FWHM* at 5.9 keV. This calculation was performed and the results are presented in Fig. 7.

The equivalent noise charge dielectric noise is given by,

1 $$EN{C}_{D}=\frac{1}{{\rm{q}}}\sqrt{{{\rm{A}}}_{2}2{\rm{k}}TDC}$$

where q is the electric charge, A_2_ is a dimensionless constant (here taken to be 1.18) that depends on the type of signal shaping^34^, k is the Boltzmann constant, *T* is the temperature (in units of K), *D* is the dielectric dissipation factor, and *C* is the capacitance. Each lossy dielectric in close proximity to the input of the preamplifier has its own dielectric noise which is dependent on its own dielectric dissipation factor and capacitance, but it is common to combine all the dielectric noise sources and state an apparent overall dielectric dissipation factor and capacitance.

**Figure 8:**
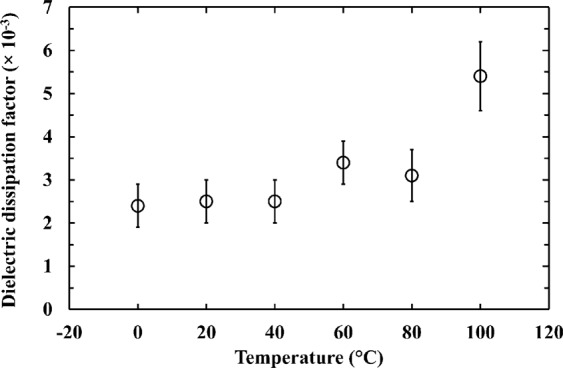
The estimated dielectric dissipation factor of Al_0.52_In_0.48_P over the temperature range of 0 °C to 100 °C.

Nevertheless, assuming that the overall dielectric noises for the two spectrometers were identical except for the different contributions from the photodiodes themselves (i.e. arising from their different capacitances) the dielectric dissipation factor of Al_0.52_In_0.48_P itself can be estimated using Equation 1 and the procedure as described in ref.^29^. As such, the dielectric dissipation factor of Al_0.52_In_0.48_P was estimated at different temperatures for the first time, and is presented in Fig. 8. The uncertainties (error bars) in the dielectric dissipation factor shown in Fig. 8 reflect the uncertainties in the capacitances of the detectors and the uncertainties in the dielectric noise which were propagated from the uncertainties in the energy resolution.

At 100 °C, the dielectric dissipation factor of the Al_0.52_In_0.48_P was estimated to be 5.4 × 10^−3^ ± 0.8 × 10^−3^. At 0 °C was 2.4 × 10^−3^ ± 0.5 × 10^−3^. The dielectric dissipation factor at 20 °C (2.5 × 10^−3^ ± 0.5 × 10^−3^) was found to be similar to the only previously reported measurement of the Al_0.52_In_0.48_P dielectric dissipation factor of at room temperature (2.2 × 10^−3^ ± 1.1 × 10^−3^)^29^.

## Discussion

The electrical characteristics and photon counting spectroscopic X-ray detection performance of Al_0.52_In_0.48_P p^+^-i-n^+^ mesa photodiodes with two different diameters (217 µm ± 15 µm and 409 µm ± 28 µm) were studied as a function of temperature, *T* (0 °C ≤ *T* ≤ 100 °C). The photodiodes are the thickest (6 μm thick i layer) Al_0.52_In_0.48_P X-ray photodiodes ever characterised for their high temperature ( > 20 °C) performance.

Measurements of detector capacitance as a function of applied reverse bias showed that the capacitances of both photodiodes were broadly consistent across the investigated temperature range when operated at high reverse bias. Measurements of detector leakage current as a function of applied reverse bias showed that the devices had low leakage currents (<5 pA) even when operated at the maximum investigated reverse bias (100 V) and temperature (100 °C).

With the detectors connected to a custom-made low-noise charge-sensitive preamplifier, ^55^Fe radioisotope X-ray source spectra were accumulated across the temperature range. At the highest investigated temperature (100 °C) and greatest reverse bias (100 V), the best energy resolutions (*FWHM* at 5.9 keV) achieved with the 217 µm and 409 µm diameter detectors were 1.31 keV ± 0.04 keV and 1.64 keV ± 0.08 keV, respectively. At the lowest investigated temperature (0 °C), these improved to 0.82 keV ± 0.04 keV and 0.95 keV ± 0.08 keV. The different noise contributors to the achieved energy resolutions were computed as functions of shaping time at different temperatures. The improvement in energy resolution as the temperature was reduced was largely due to decreased leakage current of the spectrometers with reduced temperatures. For the first time, the temperature dependence of dielectric dissipation factor of Al_0.52_In_0.48_P was estimated, a value of 5.4 × 10^−3^ ± 0.8 × 10^−3^ was found at 100 °C.

The front-end electronics is the main noise contributor to the degradation of the energy resolution of the Al_0.52_In_0.48_P spectrometers. At the optimum available shaping time (i.e. that which gave the best energy resolution), the dielectric noise was the most significant noise component among the various electronic noise components of the reported spectrometer. Dielectric noise is due to thermal fluctuations in insulators that are close to, or in contact with, the preamplifier input^32^; this includes contributions from the detector, input JFET, and any other lossy dielectrics in close proximity. Assuming dielectric dissipation factors of 2 × 10^−3^ for Si^35^ and 3 × 10^−3^ for Al_0.52_In_0.48_P (from Fig. 8) at room temperature, the dielectric noise contribution of the Al_0.52_In_0.48_P detectors (25 e^−^ rms for 217 µm diameter Al_0.52_In_0.48_P photodiode and 50 e^−^ rms for 409 µm diameter Al_0.52_In_0.48_P photodiode) and the input Si JFET (40 e^−^ rms) can be computed from Equation 1. The dielectric noises beyond those of the detector and JFET can be referred to as stray dielectric noise; such noise can be reduced by directly wire-bonding the input JFET as a bare die to the detector, rather than using a packaged JFET^28^. The stray dielectric noise (e.g. 45 e^−^ rms at 20 °C) can be estimated by the subtraction in quadrature of the dielectric noise of the Al_0.52_In_0.48_P detectors and the input Si JFET from the total dielectric noise, assuming that the noise of the feedback capacitance is small in proportion. If the stray dielectric noise could be eliminated entirely, a much better energy resolution for the spectrometers would be expected (e.g. at 20 °C, *FWHM* at 5.9 keV of 630 eV and 810 eV are expected for the 217 µm and 409 µm diameter Al_0.52_In_0.48_P photodiodes, respectively). Similar to the stray dielectric noise, elimination of the stray white series noise can further improve the energy resolution of the spectrometers. The multidimensional unconstrained nonlinear minimisation to the measured *FWHM* at 5.9 keV as a function of shaping time revealed the presence of 3 pF and 5 pF total capacitance at 20 °C in the 217 μm and 409 μm photodiode spectrometers, respectively. Considering the capacitances of the photodiodes (0.6 pF for the 217 μm diameter photodiode and 2.5 pF for the 409 μm diameter photodiode), the capacitance of the input JFET (2 pF), and assuming negligible contribution from the feedback capacitance, 0.4 pF and 0.5 pF additional stray capacitance were calculated for the 217 μm and 409 μm photodiode spectrometer, respectively. Subtracting in quadrature the known series white noise (17 e^−^ rms and 13 e^−^ rms for the 217 μm and 409 μm photodiode spectrometer, respectively) from the total series white noise (19 e^−^ rms and 15 e^−^ rms for the 217 μm and 409 μm photodiode spectrometer, respectively), the stray white series noise can be estimated; it was 8 e^−^ rms for 217 µm photodiode spectrometer and 7 e^−^ rms for the 409 µm photodiode spectrometer at 20 °C and at the optimum shaping time (2 μs for 217 µm photodiode spectrometer and 10 μs for the 409 µm photodiode spectrometer). If the stray series white noise could be also eliminated entirely, the energy resolution (*FWHM* at 5.9 keV) for Al_0.52_In_0.48_P spectrometers can be further improved to 620 eV for the 217 µm diameter photodiode and 800 eV for the 409 µm diameter photodiode at 20 °C. Even though the Fano limited energy resolution (*FWHM*_Fano_) at 5.9 keV of 4H-SiC and Al_0.52_In_0.48_P are similar (145 eV for Al_0.52_In_0.48_P cf. 160 eV for 4 H-SiC^11^), a smaller *FWHM* at 5.9 keV was obtained for the 4H-SiC detector (196 eV (corresponding to 11 e^−^ rms) at 30 °C)^11^ with respect to that found here for the Al_0.52_In_0.48_P detectors. This is thought to be mainly due to the custom ultra-low noise CMOS preamplifier (intrinsic equivalent noise charge of 3 e^−^ rms at room temperature, at a shaping time of 15 μs) to which the SiC was coupled in ref.^11^. If the 217 µm diameter Al_0.52_In_0.48_P photodiode was coupled to the same preamplifier electronics, and a 15 μs shaping time was selected, a *FWHM* at 5.9 keV as low as 360 eV (corresponding to 29 e^−^ rms) at 20 °C may be expected. The expected energy resolution was computed by adding in quadrature the Al_0.52_In_0.48_P photodiode’s white series noise (2 e^−^ rms), white parallel noise (8 e^−^ rms), dielectric noise (25 e^−^ rms), the Fano noise (12 e^−^ rms), and the electronic noise of the preamplifier (3 e^−^ rms). Such high dielectric noise is due to the high capacitance of the Al_0.52_In_0.48_P photodiode (0.6 pF for the Al_0.52_In_0.48_P photodiode cf. 0.1 pF for the 4H-SiC detector at room temperature^11^) as well as the high dielectric dissipation factor of the material (3 × 10^−3^ for Al_0.52_In_0.48_P cf. 4 × 10^−4^ for 4H-SiC at room temperature^36^).

The results show that the Al_0.52_In_0.48_P photodiodes had a low leakage current even at high temperature (100 °C) and that they can be used as high temperature tolerant X-ray detector for high-temperature X-ray photon counting spectroscopy. Such instrumentation is required for future space applications, including planetary investigation X-ray fluorescence spectroscopy and Solar X-ray monitoring, as well as extreme environment terrestrial applications.

## Methods

### Device design

The epilayer structures were grown nearly lattice matched on a commercial (100) GaAs n^+^ substrate by metalorganic vapour phase epitaxy (MOVPE). The epitaxial surface of the GaAs substrate had a miscut angle of 10° towards the <111> A. A 0.1 µm n type (Si dopant) Al_0.52_In_0.48_P layer was grown on the GaAs substrate, followed by a 6 µm thick unintentionally doped layer, and then a 0.2 µm p type (Zn dopant) layer. A 0.01 µm GaAs p layer was grown as a cap on top of the Al_0.52_In_0.48_P p type layer. The doping density of the p type and n type Al_0.52_In_0.48_P layers were 5 × 10^17^ cm^−3^ and 2 × 10^18^ cm^−3^, respectively. Initially, a 1:1:1 H_3_PO_4_:H_2_O_2_:H_2_O solution was used to chemically etch the circular mesa photodiodes of two different diameters. However, due to a slow vertical etching rate, the etching solution was changed to 1:1:1 K_2_Cr_2_O_7_:HBr:CH_3_COOH solution. This was followed by a 10 s finishing etch in a 1:8:80 H_2_SO_4_:H_2_O_2_:H_2_O solution. After fabrication, the diameters of the devices were measured to be 217 µm ± 15 µm and 409 µm ± 28 µm, respectively, using an optical microscope. The stated uncertainties resulted from the accuracy of the optical microscope calibration. The top (p layer) contacts of the devices were Ohmic Ti/Au (20 nm/200 nm), the contact areas were 0.014 mm^2^ and 0.041 mm^2^ for the 217 µm and 409 µm diameter photodiodes, respectively. The rear planar contacts (applied to the back of the substrate) were Ohmic InGe/Au (20 nm/200 nm). The devices were gold-ball wire-bonded in a TO-5 package.

## Data Availability

Data underlying this work are contained within the paper, requests for further access to any information may be addressed to the authors.
